# Association between High Ambient Temperatures and Road Crashes in an Australian City with Temperate Climate: A Time-Series Study, 2012–2021

**DOI:** 10.3390/ijerph20116000

**Published:** 2023-05-30

**Authors:** Yannan Li, Blesson Mathew Varghese, Jingwen Liu, Peng Bi, Michael Tong

**Affiliations:** 1 School of Public Health, The University of Adelaide, Adelaide, SA 5005, Australia; 2 National Centre for Epidemiology and Population Health, ANU College of Health and Medicine, The Australian National University, Canberra, ACT 2601, Australia

**Keywords:** high temperature, road crash, accident, climate change, Australia

## Abstract

(1) Background: High ambient temperatures are associated with increased morbidity and mortality rates, and some evidence suggests that high temperatures increase the risk of road crashes. However, little is known regarding the burden of road crashes attributable to no-optimal high temperatures in Australia. Therefore, this study examined the effects of high temperatures on road crashes using Adelaide in South Australia as a case study. (2) Methods: Ten-year daily time-series data on road crashes (n = 64,597) and weather during the warm season (October–March) were obtained between 2012 and 2021. A quasi-Poisson distributed lag nonlinear model (DLNM) was used to quantify the cumulative effect of high temperatures over the previous five days. The associations and attributable burden at moderate and extreme temperature ranges were computed as relative risk (RR) and attributable fraction. (3) Results: There was a J-shaped association between high ambient temperature and the risk of road crashes during the warm season in Adelaide, and pronounced effects were observed for minimum temperatures. The highest risk was observed at a 1 day lag and lasting for 5 days. High temperatures were responsible for 0.79% (95% CI: 0.15–1.33%) of road crashes, with moderately high temperatures accounting for most of the burden compared with extreme temperatures (0.55% vs. 0.32%). (4) Conclusions: In the face of a warming climate, the finding draws the attention of road transport, policy, and public health planners to design preventive plans to reduce the risk of road crashes attributable to high temperatures.

## 1. Introduction

Climate change has resulted in increased temperatures [1] and increases in the frequency, severity, and duration of heatwaves worldwide [2,3,4]. Previous studies have reported that high temperature, as one of the well-documented human health hazards, is associated with an increased risk of morbidity and mortality [5,6,7,8,9,10]. According to the Global Burden of Disease study, high-temperature exposure has led to the loss of 308,000 lives and resulted in a reduction of 11.7 million years of healthy life worldwide in 2019 [11].

Road crashes due to high temperature have been identified as one of the major public health concerns given their significant contribution to unintentional mortality and morbidity worldwide, particularly in the context of climate change [12]. According to the World Health Organization Road Safety report, there are 20 to 50 million nonfatal road crashes annually, leading to approximately 1.24 million deaths [13]. A global analysis reported that the deaths due to road crashes attributable to high temperatures significantly increased from 20,270 in 1990 to 28,396 in 2019 [14]. A growing body of evidence has shown that high ambient temperatures are associated with an increased risk of road traffic crashes [15,16,17,18,19,20]. For example, a study in China reported that significantly more road crashes occurred during summer [17]. An Italian study found a positive association between high temperatures and road crashes (indicating a relative risk of 1.12 (95% CI: 1.09–1.16) for high temperature (99th percentile vs. 75th percentile of daily mean temperature) [16]. A recent nationwide investigation conducted in Spain utilized a more recent and expansive dataset [20] to corroborate its prior findings from a regional analysis carried out in Catalonia, Spain [21]. Both articles showed that high ambient temperatures were associated with an increased risk of road crashes [20,21]. Moreover, a study in the United States showed that road fatalities increased by 3.4% on heatwave days compared with nonheatwave days [22].

The higher incidence of road crashes during hot days might be due to high temperatures, which can affect human physical and cognitive performance [20,21], road conditions [23], and vehicle performance [24], leading to an increase in the number of road crashes. While studies have explored this association, the majority of them have been conducted in the northern hemisphere, where the climatic conditions are different, and epidemiological characteristics are dissimilar from those in the southern hemisphere. For example, Catalonia in Spain and Adelaide in Australia both have a Mediterranean climate, with similar average summer temperatures of 28–29 °C and 26–29 °C, respectively [25,26]. However, Adelaide experiences considerable temperature variations during summer, with a number of days reaching over 40 °C in a year and the night-time temperatures exceeding 30 °C [27]. Furthermore, there are an average of 64.1 road crashes per day in Catalonia, Spain [28] but only 35.4 road crashes per day in Adelaide, Australia [29]. Given the diverse climatic and epidemiological characteristics and a lack of evidence of the association between temperature and road crashes in Australia, it is imperative to examine the effects of rising ambient temperatures on road crashes in the context of climate change. Without a thorough understanding of this relationship, it will be difficult to develop effective strategies to prevent or mitigate the adverse impacts of climate change on road safety. Therefore, conducting research in this area is crucial to establish a robust evidence base for policymakers, road safety authorities, and drivers. This may enable the refinement of traffic guidelines and codes of practice (e.g., early warning messages to drivers), thereby reducing road accidents in the context of climate change.

This study aimed to investigate the association between high ambient temperatures and road crashes during warm seasons and quantify the attributable burden of road crashes due to high temperatures in an Australian city as a case study.

## 2. Materials and Methods

### 2.1. Study Area

Adelaide, the capital city of South Australia, is one of the most populous cities in Australia, with a population of approximately 1.4 million (2021 census) living in an area of 3260 km^2^ [30]. Adelaide has a Mediterranean climate with mild and wet winters and hot and dry summers [31]. The annual average temperature ranges from 12.3 °C to 22.5 °C [25].

### 2.2. Data Sources

#### 2.2.1. Road Traffic Crash Records

The 10-year data on road crashes in Adelaide from 1 January 2012, to 31 December 2021, were obtained from the Road Crash Database (Traffic Accident Register (TARS)) of the Department of Planning, Transport, and Infrastructure (DPTI), Government of South Australia [29]. Each road crash record in the TARS dataset included information on the date and time of the road crash, crash type (hit fixed object, rear end, right angle, etc.), location, and casualty type: minor, serious, or fatal injury. Further details on the TARS dataset and a complete list of variables can be found in the DPTI metadata [29]. For this study, road crashes were aggregated into a time-series format of the daily number of road crashes.

#### 2.2.2. Meteorological Data

Daily meteorological data were obtained from the Scientific Information for Land Owners (SILO) interpolated climate raster datasets hosted by the Science and Technology Division of the Queensland Government’s Department of Environment and Science [32]. Meteorological data, including daily minimum temperatures (Tmin), daily maximum temperatures (Tmax), daily relative humidity, daily solar radiation, and daily rainfall during the study period (2012–2021), were downloaded from the SILO website (https://www.longpaddock.qld.gov.au/silo/, accessed on 16 March 2023). Daily mean temperatures (Tmean) were calculated as the average of Tmin and Tmax. These raster meteorological data are based on a 0.05° × 0.05° grid cell (approximately 5 km × 5 km resolution) that is spatially interpolated from the Australian Bureau of Meteorology weather stations. The meteorological data from all grids covering the Adelaide metropolitan area (defined using postcodes: 5000–5174) were averaged to represent the weather conditions in Adelaide.

### 2.3. Study Design and Statistical Analysis

A time-series study design was used to investigate the association between ambient temperatures (Tmin, Tmean, and Tmax) and road crashes in Adelaide. A quasi-Poisson regression model (accounting for the overdispersion of road crash counts) combined with a distributed lag nonlinear model (DLNM) was used to explore the nonlinear and lagged effect of ambient temperature on road crashes [33,34]. We selected 5 days as the maximum number of lag days in the model based on the combination of Akaike’s information criterion (AIC) and previous studies [35,36,37]. As the focus of the study was heat-related road crashes, we restricted the analysis to the warm season in Australia (October to March).

Seasonality was adjusted for using a natural cubic spline with 4 degrees of freedom (df) of the day of the year, and an interaction between this spline term and year allowed for different seasonal trends across the study period [38,39,40,41]. In order to control long-term trends, the model also included a natural spline function of time with approximately 1 df for every 10 years. A natural cubic spline with 2 knots (at the 50th and 90th percentile of temperature) placed at equally spaced values on the log scale was used to flexibly model the lag–response curve [33,42]. In addition, we controlled for a set of potential confounders such as the day of the week, public holidays, school holiday periods (Term 1: mid-April–end of April; Term 2: early July–late July; Term 3: end of September–mid-October; Term 4: mid-December–end of January) [43], and the days immediately preceding or following a public holiday. We controlled for other meteorological factors including relative humidity, solar radiation, and rainfall using natural cubic splines (ns) with 3 degrees of freedom each [44], as these meteorological variables are potential confounders of the association between ambient temperatures and road crashes [45]. Model fits were evaluated using Akaike’s information criterion for quasi-Poisson (Q-AIC), and the model with the lowest Q-AIC value was chosen as the final model [33,42].

The quasi-Poisson DLNM model used for analysis can be illustrated as follows:

(1) $$Yt\sim Poisson\;\left({µ}_{t}\right)\;\begin{array}{ll} Log\left(\mu_{t}\right)=\alpha + & cb\left(Temp_{t},\;5\right)+ns\left(doy,\;4\;df\right):factor\left(year\right)\\ & +ns\left(time,1\;df/10\right)+\beta_{1}\times dow_{t}+\beta_{2}\times public\;holiday_{t}\\ & +\beta_{3}\times school\;holiday_{t}+\beta_{4}\times begin\;end_{t}\\ & +ns\left(radiation_{t},3\;df\right)+ns\left(rainfall_{t},3\;df\right)\\ & +ns\left(humidity_{t},\;3\;df\right)+offset\left(log\left(population_{t}\right)\right)+E_{t} \end{array}$$

where *Yt* is the time-series outcome; *t* is the day of the study period; *µ_t_* is the expected count of road crashes on day *t*; *α* is the intercept; *cb* (*Temp_t_*, *5*) is the cross-basis function for ambient temperatures (Tmin/Tmean/Tmax) with both the response and lag dimension applied from the DLNM, and 5 was the maximum lag days used; *ns(doy,4 df):factor( year)* is the natural spline function of day of the year with 4 degrees of freedom (df) controlling seasonality and long-term trends; *dow_t_* is the day of the week on day *t*; *public holiday_t_*, *school holiday_t_*, and *begin end_t_* and are binary variables for adjusting public holidays, school holidays, and the days immediately preceding or following a public holiday, respectively; *ns* (*radiation_t_*, *3 df*), *ns* (*rainfall_t_*, *3 df*), and *ns* (*humidity_t_*, *3 df*) are the natural cubic spline functions with 3 degrees of freedom controlling for solar radiation, rainfall, and relative humidity, respectively; *log* (*population_t_*) is the log scaled population of Adelaide added in the model as an offset; *E_t_* is the model residual.

The high temperature–road crash association is reported as relative risk (RR) with a 95% confidence interval (CI) at moderately high (90th percentile) temperature and extremely high (99th percentile) temperature compared with the minimum road crash temperature (MCT). The MCT represents the temperature at which the risk of road crashes is the lowest and was derived from the overall cumulative exposure–response relationship. We calculated the risk of road crashes with high ambient temperatures at individual and accumulated lag days, representing the delayed and overall cumulative effects, respectively.

Road crashes attributable to high ambient temperatures, including attributable fraction (AF) and attributable number (AN), were calculated using the backward perspective method of Gasparrini et al. [46,47]. These two measures (AN and AF) represent the proportion and number of road crashes attributed to high ambient temperatures, respectively. The high ambient temperatures were further divided into ranges of moderately and extremely high temperatures using the temperature percentiles defined above as cutoff points.

Sensitivity analyses were conducted by modifying the modeling choices (changing the df for the time and using different lag days) to test the robustness of the results. All the statistical analyses were performed using R (version 4.0) using packages such as ‘dlnm’ and ‘gnm’ and the function ‘attrdl’ [33].

## 3. Results

### 3.1. Descriptive Statistics

There were a total number of 64,597 road crashes during the warm season (October–March) over the study period of 2012–2021 in the Adelaide Metropolitan Area. The daily mean number of road crashes was 35.4 (range: 5–111). The descriptive statistics for the meteorological variables are summarized in Table 1. The average daily Tmin, Tmean, and Tmax were 13.8 °C (range: 4.0–30.0 °C), 20.1 °C (range: 8.5–37.0 °C), and 26.4 °C (range: 12.0–46.0 °C), respectively. The average daily relative humidity (rh) was 57.1% (range: 12.0–96.0%); the average daily solar radiation was 22.7 MJ m^−2^ (range: 5.0–33.0 MJ m^−2^); the average daily rainfall was 0.8 mm (range: 0.0–75.2 mm). The time-series plots for daily road crashes and the meteorological variables are presented in Supplementary Figure S1.

### 3.2. Exposure–Response Relationship

**Figure 1:**
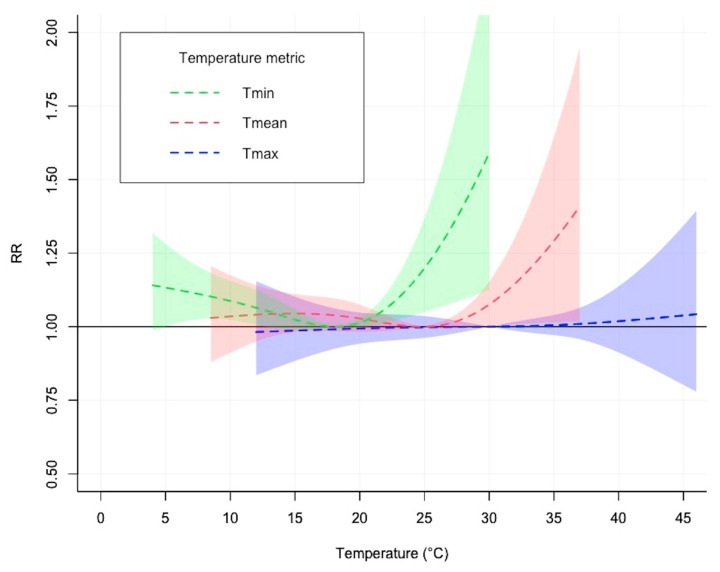
The cumulative exposure–response relationship between different temperature metrics (Tmin, Tmean, and Tmax) and road crashes compared with MCT for 5 lag days in Adelaide. The dark-color dotted lines represent RR, and the light-color shaded regions represent 95% CI. Tmin: daily minimum temperature; Tmean: daily mean temperature; Tmax: daily maximum temperature; RR: relative risk.

**Figure 2:**
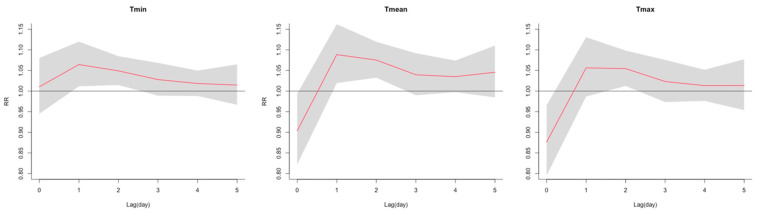
The estimated lag–response associations of extremely high temperatures (Tmin, Tmean, and Tmax at 99th percentile) and road crashes compared with MCT; the 95% CIs are shown in grey areas, and the RRs are shown as red lines. Tmin: daily minimum temperature; Tmean: daily mean temperature; Tmax: daily maximum temperature; RR: relative risk.

Figure 1 illustrates the cumulative exposure–response relationship between daily temperature (Tmin, Tmean, Tmax) and road crashes cumulative over lag days 0–5. A nonlinear association was observed for each temperature metric. The trends in the overall cumulative exposure–response relationships between different temperature metrics and road crashes were similar. Specifically, the exposure–response relationship exhibited a J-shaped curve, with a substantially increased risk of road crashes when the daily minimum temperature exceeded 18.1 °C and the daily mean temperature exceeded 24.8 °C. However, there was a less significant increase in the risk of road crashes with increases in the daily maximum temperature. Figure 2 shows the effects of heat on road crashes along the lags for extremely high ambient temperatures at the 99th percentile (Tmin: 25 °C, Tmean: 33 °C, and Tmax: 42 °C). The effects of extremely high temperatures on road crashes peaked at a lag of 1 day, followed by a cumulative effect until a lag of 5 days. Three-dimensional plots of the overall exposure–lag–response associations and the overall cumulative exposure–response relationships with temperature distributions (Tmin, Tmean and Tmax) can be found in Supplementary Materials (Figures S2–S4). The results of the quasi-Poisson DLNM estimation of the temperature distributions (Tmin, Tmean, and Tmax) and road crashes can be found in Supplementary Material (Tables S1–S3).

The calculated cumulative effects of different temperature metrics (Tmin, Tmean, and Tmax) on the road crashes are shown in Table 2. Across the different temperature metrics, higher RRs were observed for Tmin than for Tmean and Tmax. The cumulative RR (lag 0–5 days) for Tmin during moderately high temperatures (90th percentile; 20.0 °C vs. 18.1 °C) was 1.013 (95% CI: 0.996–1.030), which then further increased to 1.198 (95% CI: 1.049–1.369) at extremely high temperatures (99th percentile; 25.0 °C vs. 18.1 °C) (Table 2). The cumulative RR (lag 0–5 days) for Tmean during moderately high temperatures (90th percentile; 27.0 °C vs. 24.8 °C) was 1.012 (95% CI: 0.994–1.030), which further increased to 1.168 (95% CI: 1.010–1.351) during extremely high temperature (99th percentile; 32.5 °C vs. 24.8 °C). However, the cumulative RR (lag 0–5 days) for Tmax during moderately and extremely high temperatures did not show a significantly increased risk of road crashes compared with the MCT.

### 3.3. Attributable Risk of Road Crashes Due to High Ambient Temperature

The estimates of AN and AF for road crashes attributed to nonoptimal temperatures are depicted in Table 3. Overall, high ambient temperatures were responsible for 0.16–0.79% (corresponding AN is 105–513) of the road crash burden. Compared with the three-temperature metrics, Tmin and Tmean were associated with a higher burden of road crashes than Tmax, in particular due to moderately high temperatures. The moderately high temperatures accounted for 0.55% of the road crash burden in Tmin, compared with 0.32% of the extremely high temperatures using the temperature metric Tmin, and the moderately high temperatures accounted for 0.47% of the road crash burden in Tmean, compared with 0.22% of the extremely high temperatures using the temperature metric Tmean. However, the attributable road crashes due to Tmax were not statistically significant.

### 3.4. Sensitivity Analysis

The results of the sensitivity analyses involving varying the maximum lag days from three to seven (Figure S5) and changing the df of the natural cubic spline for the calendar year from four to nine per year (Figure S6) indicated that the results were robust and not dependent on the modeling assumptions. The residuals for the DLNM models followed a normal distribution, and no significant autocorrelations were found in the residuals (Figure S7).

## 4. Discussion

This study used the DLNM approach and found a J-shaped association between high ambient temperatures and the risk of road crashes during the warm season in Adelaide, which indicated that there was an MCT with the lowest road crash risk, and high ambient temperatures above the MCT increased the risk of road crashes. Our analysis revealed that the highest relative risk of road crashes during high temperatures occurred when using the daily minimum temperature. The effect of high temperature on road crashes was acute, with the risk peaking at a lag of 1 day and then gradually decreasing until a lag of 5 days. The burden of road traffic crashes was mainly attributable to moderately high temperatures, with relatively less contribution from extremely high temperatures.

This study found that high ambient temperatures resulted in increased numbers of road crashes, which is consistent with the results of previous studies from other countries in the northern hemisphere [15,16,19,48,49,50]. For example, a study in Shenzhen, China, reported that hourly road traffic casualties increased by 0.9% for each 1 °C increase above the optimal temperature of 17 °C [51]. A study in Catalonia, Spain, reported that the road crash risk increased by 2.9% (95% CI: 0.7–5.1%) during heat wave days [21]. Moreover, it is worth noting that the daily number of road crashes in Catalonia in summer was 64.1 for a 7.1 million population [21] compared with 35.4 crashes for a 1.4 million population in Adelaide. The comparison further highlighted the higher road crash rates in Adelaide during the summer period. A meta-analysis study also found a significant association between high temperatures and road crashes (pooled RR = 1.025) [51].

The mechanism linking high temperatures to road crashes is complex, with many contributing factors, such as human behavior, vehicle conditions, and environmental factors [52]. Higher temperatures could lead to reduced vigilance, thus directly resulting in poor driving performance [53,54,55,56], such as larger steering adjustment, deviating from the traffic lane, and missing traffic signals, thereby finally increasing the risk of road crashes in high-temperature environments [57,58]. Furthermore, there is a possible biological mechanism through which high ambient temperatures cause 5-hydroxytryptamine (5-HT) dysfunction, which adversely affects decision-making ability [59], thereby increasing the risk of road crashes. Drivers should be reminded to pay attention to high ambient temperatures before traveling during increasingly hot days in the context of climate change in Australia. Regarding vehicle and environmental factors, high ambient temperatures can affect vehicle and road conditions, leading to excessive tire pressure, softening the road surface, and damaging road structure [60], which may increase the risk of road crashes. The maintenance of high vehicle standards by car owners and regular checking of road conditions by infrastructure agencies could be helpful in effectively reducing road crashes to some extent due to high ambient temperatures, particularly in South Australia, where, generally, there is no compulsory annual inspection for privately owned vehicles [61].

Our study found a stronger effect of heat on road crashes by using the daily minimum temperature than with using daily mean and maximum temperatures. Several studies have observed a large increase in daily minimum temperature in high-latitude areas over the past few decades [62,63]. The daily minimum temperature can reflect night temperatures, which can affect the quality of sleep before the day of traveling [64]. A growing number of studies has reported the harmful effects of hot nights on human health [64,65,66,67]. Hot nights can cause prolonged thermal stress and sleep disturbances, increase cumulative fatigue and the risk of driving during the daytime, and finally result in road crashes [68,69,70]. However, the majority of the previous studies have explored the exposure-response association between the ambient temperature and road crashes using the daily mean and maximum temperatures [16,22,50], and limited studies have used the daily minimum temperature. A Spanish study reported that there was no significant association between the daily minimum temperature and road crashes [21], which differs from the findings presented here. This disparity could be explained by the following reasons: (1) The methods employed in assigning weather values to the location of road crashes were different. Basagaña et al. employed the average daily minimum temperature of multiple weather stations within each climatic region [21], whereas our approach involved the use of gridded weather data. The application of different methods for assigning weather values may produce different estimates of the weather conditions at the site of road crashes, which could affect the estimated association between daily minimum temperature and road crashes. (2) Different cutoff points were used for calculating RRs. Our study used the 99th percentile of the daily minimum temperature to calculate the RRs of road crashes, while Basagaña et al. used the average minimum temperature of multiple weather stations within each climatic region to calculate the climatic region-specific RRs of road crashes and then used a random effects meta-analysis to obtain the overall RR [21]. Therefore, our study found a specific range of high minimum temperatures that were associated with an increased risk of road crashes, which was not captured in Basagaña et al.’s study. This may imply that studies in different countries with various climate patterns are needed to broadly explore the impact of the daily minimum temperature on road crashes. To further explore the lag effects of ambient temperature on road crashes, we found that high ambient temperatures had an acute effect on road crashes, and the risk of road crashes peaked at a lag of 1 day, with an effect lasting for 5 days. Our findings are in accordance with those of previous studies indicating that the effect of high temperatures on road crashes was immediately apparent [19] and the cumulative effects could last for up to 7 days in Mediterranean climates and humid continental climates [20,49]. A possible explanation for the findings might be the lag effect of the ambient temperature on human health. For example, the mortality among vulnerable populations and hospital admissions caused by mental health disorders, renal diseases, respiratory diseases, and cardiovascular diseases would continue to increase after heatwaves [71,72,73,74]. This suggests that the health effects of high ambient temperatures can persist for several days, continue to affect drivers, and lead to the lag effect of ambient temperature on road crashes.

Furthermore, this study demonstrated that the majority of road crashes were more attributable to moderately high temperatures than to extremely high temperatures. No previous studies have reported the attributable burden of road crashes due to either moderately or extremely high temperatures. Several other studies in Australia and China have indicated that a greater burden of cause-specific mortality can be more attributed to moderately high temperatures than to extremely high temperatures [47,75,76]. The main reason for these similar results could be the higher frequency of moderately high temperatures than extremely high temperatures across the years. Other potential reasons could include changed behaviors during extremely hot days and temporal misalignment between exposure and outcome; for instance, residents and drivers are encouraged and alerted to take necessary actions to prevent heat exposure during extremely high temperatures such as using car air conditioning systems and reducing outdoor activities. Another possible reason could be the temporal misalignment between exposure and outcome. The daily maximum temperatures usually occur in the late afternoon between 2 and 4 p.m., while the peak traffic occurs at 7–9 a.m. and 4–6 p.m. in Adelaide [77].

This is the first study to investigate the nonlinear and lag relationship between high ambient temperatures and road crashes in Australia. This study examined the effects of high ambient temperatures on road crashes and quantified the attributable burden of road crashes due to high ambient temperatures. Moreover, using fine-resolution gridded weather data (5 km × 5 km) to assign local daily temperatures to each road crash helped overcome the exposure misclassification that occurs due to using single or multiple meteorological stations’ data and allowed the better control of the temporal and seasonal patterns occurring in each region [78]. We used 10-year meteorological and road crash data and adjusted for other meteorological variables including daily relative humidity, solar radiation, and rainfall.

Several limitations of this study should be acknowledged. First, this study was conducted using a single city’s data, limiting the generalizability of the findings to other cities and regions. Subsequent studies are warranted to explore the relationship between high ambient temperatures and road crashes in other areas of Australia. Second, daily traffic volume can affect the number of road crashes. However, due to data availability, traffic volume data were not included in the modeling. Third, given the nature of observational studies, the impact of unobserved confounders that may change the association cannot be ruled out. However, such effects are considered not significant because our results were adjusted for known confounders, including the day of the week, public holidays, school holiday periods, the days immediately preceding or following a public holiday, and other meteorological factors to minimize this risk. Lastly, other individual-level risk factors may affect the association between high ambient temperatures and road crashes, such as driver health condition, driving habits, and air conditioner utilization. Future studies may consider addressing these limitations and projecting the future burden of road crashes due to increasing temperature in the context of climate change, which could help health departments and transportation agencies optimize resource allocations and road safety guidelines.

The research findings from this study have important policy implications. Due to the lag effect of high ambient temperatures on road safety, it is important to issue road safety warnings in advance to reduce road crashes. Furthermore, this study highlights the adverse effects of high daily minimum temperatures and hot nights, emphasizing the need for greater attention toward night-time weather conditions and road safety measures.

## 5. Conclusions

This study found that high ambient temperatures were associated with an increased occurrence of road crashes in Adelaide, and high daily minimum temperatures had a stronger heat effect on road crashes. The effects of high temperatures on road crashes were the most prominent at a lag of 1 day and persisted for up to 5 days. Approximately 1.61% of road crashes could be attributed to high ambient temperatures, with moderately high temperatures accounting for more road crashes than extremely high temperatures. Future research could focus on cities with different demographic characteristics and climatic conditions, considering traffic volume, individual-level risk factors, and heat exposure. Given the projected increase in global temperature with more frequent hot days, road crashes attributed to high temperatures are estimated to rise in the future. The findings highlight the significant impact of temperature on road crashes and suggest that it is imperative to develop preventive measures and raise driver awareness of road safety on hot days.

## Figures and Tables

**Table 1:** Descriptive statistics of meteorological data in warm season (October–March) in Adelaide, 2012–2021 (1823 days).

Variable	Percentile						
	Min	Mean	Max	50th	75th	90th	99th
Tmin (°C)	4.0	13.8	30.0	13.0	16.0	20.0	25.0
Tmean (°C)	8.5	20.1	37.0	19.5	23.0	27.0	32.5
Tmax (°C)	12.0	26.4	46.0	25.0	31.0	35.0	42.0
Relative humidity (%)	12.0	57.1	96.0	60.0	68.0	74.0	85.0
Solar radiation (MJ m^−2^)	5.0	22.7	33.0	24.0	28.0	30.0	32.0
Rainfall (mm)	0.0	0.8	75.2	0.0	0.0	1.6	16.4

Tmin: daily minimum temperature; Tmean: daily mean temperature; Tmax: daily maximum temperature.

**Table 2:** Relative risk of different temperature metrics (Tmin, Tmean, and Tmax) for road crashes over multiple lag days in Adelaide, 2012–2021 (RR with 95% CI).

Temperature ^a^	Lag Days	Moderate High Temperature (90th Percentile) ^b^	Extreme High Temperature (99th Percentile) ^c^
Tmin	0–1	1.005 (0.994–1.016)	1.075 (0.995–1.162)
	0–2	1.007 (0.995–1.019)	1.128 (1.031–1.235)
	0–3	1.008 (0.994–1.022)	1.160 (1.045–1.287)
	0–4	1.010 (0.994–1.025)	1.181 (1.048–1.331)
	0–5	1.013 (0.996–1.030)	1.198 (1.049–1.369)
Tmean	0–1	1.004 (0.991–1.019)	0.987 (0.900–1.082)
	0–2	1.007 (0.993–1.021)	1.052 (0.951–1.165)
	0–3	1.009 (0.993–1.025)	1.089 (0.970–1.223)
	0–4	1.010 (0.993–1.028)	1.123 (0.984–1.281)
	0–5	1.012 (0.994–1.030)	1.168 (1.010–1.351)
Tmax	0–1	1.010 (0.986–1.035)	0.922 (0.831–1.025)
	0–2	1.008 (0.982–1.035)	0.975 (0.869–1.093)
	0–3	1.007 (0.978–1.036)	0.998 (0.876–1.136)
	0–4	1.005 (0.974–1.038)	1.012 (0.873–1.172)
	0–5	1.005 (0.971–1.040)	1.026 (0.871–1.209)

Tmin, daily minimum temperature; Tmean, daily mean temperature; Tmax, daily maximum temperature. ^a^ All temperatures were compared with the minimum crash temperature (MCT) of 18.1 °C (Tmin), 24.8 °C (Tmean), and 30.0 °C (Tmax). ^b^ The 90th percentile of temperature (Tmin: 20.0 °C; Tmean: 27.0 °C; Tmax: 35.0 °C). ^c^ The 99th percentile of temperature (Tmin: 25.0 °C; Tmean: 32.5 °C; Tmax: 42.0 °C).

**Table 3:** Attributable risk of high ambient temperatures for road crashes, contributed by extremely and moderately high temperatures with 95% empirical confidence intervals in Adelaide, 2012–2021.

	Tmin		Tmean		Tmax	
	AN	AF (%)	AN	AF (%)	AN	AF (%)
Heat	513 (152–877)	0.79 (0.15–1.33)	429 (18–825)	0.66 (0.04–1.28)	105 (−443–683)	0.16 (−0.68–1.02)
Moderate heat	356 (77–612)	0.55 (0.12–1.01)	308 (4–639)	0.47 (0.02–0.95)	5 (−46–61)	0.01 (−0.08–0.08)
Extreme heat	207 (58–351)	0.32 (0.08–0.53)	143 (2.4–628)	0.22 (0.02–0.40)	100 (−51–57)	0.16 (−0.67–0.98)

Tmin, daily minimum temperature; Tmean, daily mean temperature; Tmax, daily maximum temperature; AN: attributable number; AF: attributable fraction; moderate heat, temperature between MCT and 99th percentile; extreme heat, temperature above the 99th percentile. The 99th temperature percentiles are used as cutoffs for moderately and extremely high temperatures.

## Data Availability

All data used in this study are publicly available data and can be obtained from the Department of Planning, Transport, and Infrastructure (DPTI) of the Government of South Australia and the Scientific Information for Land Owners (SILO).

## Supplementary Materials

The following supporting information can be downloaded at: https://www.mdpi.com/article/10.3390/ijerph20116000/s1, Figure S1: Time-series plots for daily road crashes and meteorological variables in Adelaide, 2012–2021 (both cold and warm seasons); Figure S2: Three-dimensional plot of relationships between daily road crashes and daily minimum temperature in Adelaide, 2012–2020 (left), and the overall cumulative exposure–response associations with minimum temperature distributions (right). RR is the relative risk for road crashes; Figure S3: Three-dimensional plot of relationships between daily road crashes and daily mean temperature in Adelaide, 2012–2020 (left), and the overall cumulative exposure-response associations with mean temperature distributions (right). RR is the relative risk for road crashes; Figure S4: The three-dimensional plot of relationships between daily road crashes and daily maximum temperature in Adelaide, 2012–2020 (left), and the overall cumulative exposure–response associations with maximum temperature distributions (right). RR is the relative risk of road crashes; Figure S5: Overall cumulative exposure–response curve between temperatures (Tmin, Tmean, and Tmax) and road crashes when changing df from 4 to 9 in Adelaide, South Australia, 2012–2021. RR is the relative risk of road crashes; Figure S6: Overall cumulative exposure–response curve between temperatures (Tmin, Tmean, and Tmax) and road crashes when changing the maximum lag days from 3 to 7 days in Adelaide, South Australia, 2012–2021. RR is the relative risk of road crashes; Figure S7: Histogram, scatter plot, autocorrelation function (ACF), and partial autocorrelation function (PACF) plots of residuals using (a) Tmin, (b) Tmean, and (c) Tmax derived from DLNM for road crashes in Adelaide, South Australia, 2012–2021. Table S1: Results of the quasi-Poisson DLNM analysis of Tmin and road crashes in Adelaide, South Australia, 2012–2021. Table S2: Results of the quasi-Poisson DLNM analysis of Tmean and road crashes in Adelaide, South Australia, 2012–2021. Table S3: Results of the quasi-Poisson DLNM analysis of Tmax and road crashes in Adelaide, South Australia, 2012–2021.
