# Supplementary material for: Association between High Ambient Temperatures and Road Crashes in an Australian City with Temperate Climate: A Time-Series Study, 2012–2021
Source: Int J Environ Res Public Health. 2023 May 30;20(11):6000. doi: 10.3390/ijerph20116000 (PMC10252869; doi:10.3390/ijerph20116000)
Supplement: Supplementary file 1 [file ijerph-20-06000-s001.zip › ijerph-2316780-supplementary.pdf]

Supplementary Materials

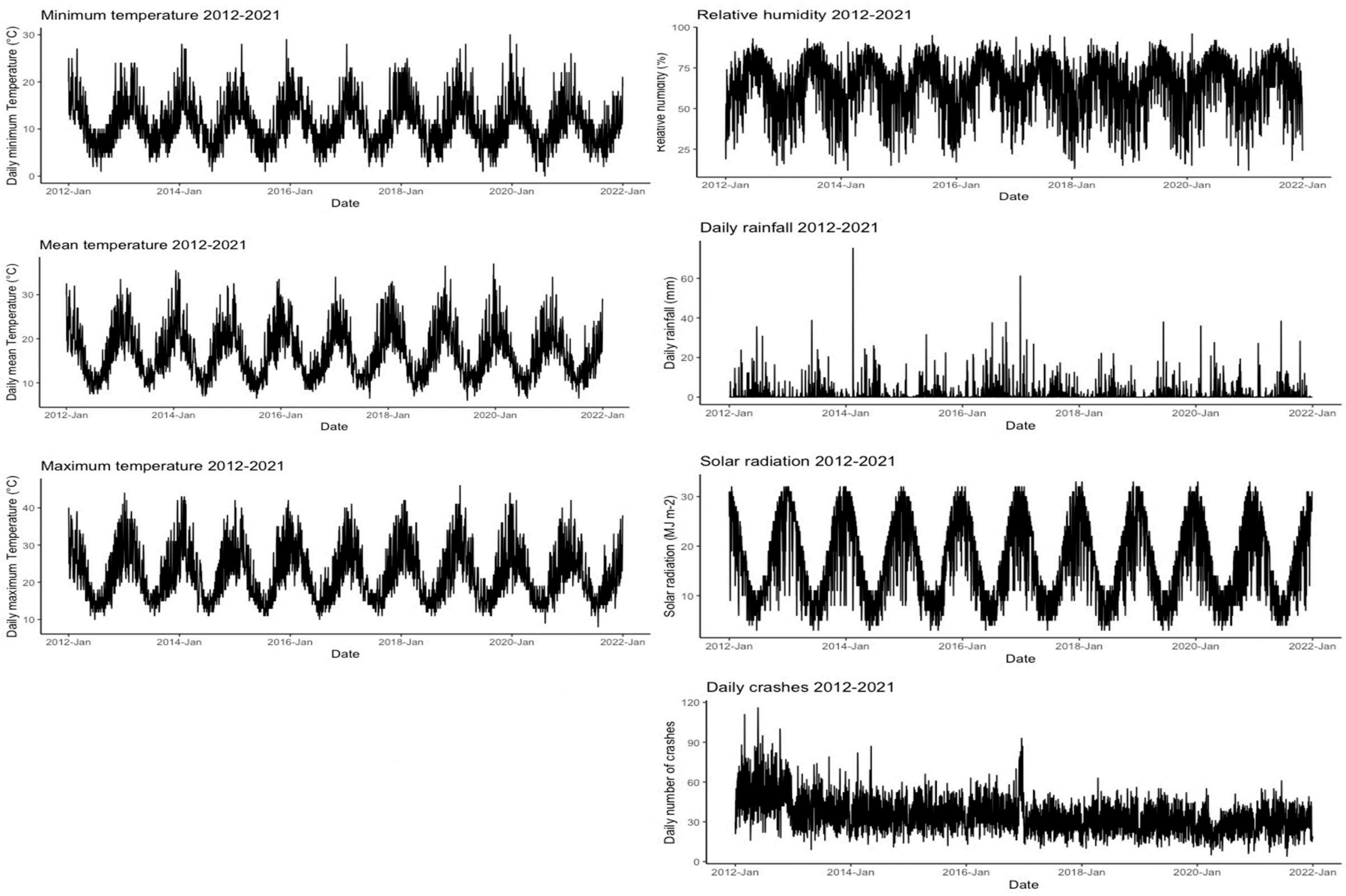

Figure S1. Time-series plots for daily road crashes and meteorological variables in Adelaide, 2012–2021 (Both cold and warm season).

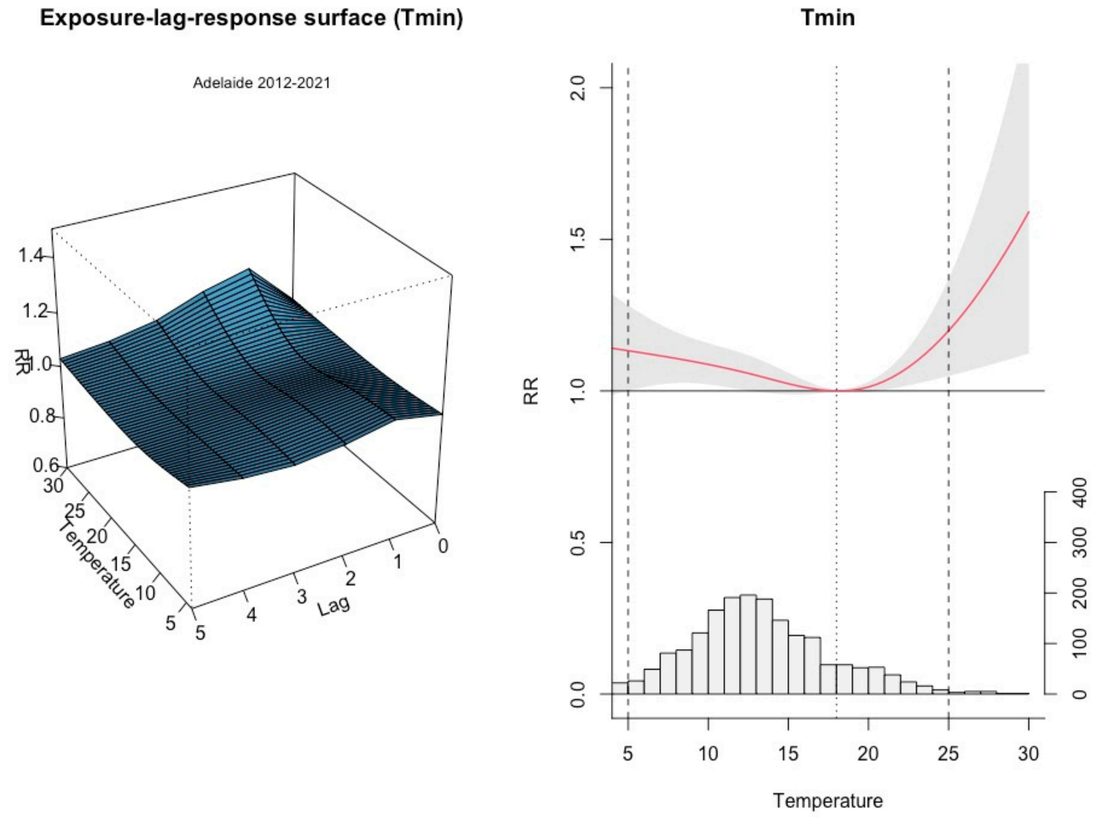

Figure S2. The three-dimensional plot of relationships between daily road crashes and daily minimum temperature in Adelaide, 2012–2020 (left), and the overall cumulative exposure-response associations with minimum temperature distributions (right). RR is the relative risk for road crashes.

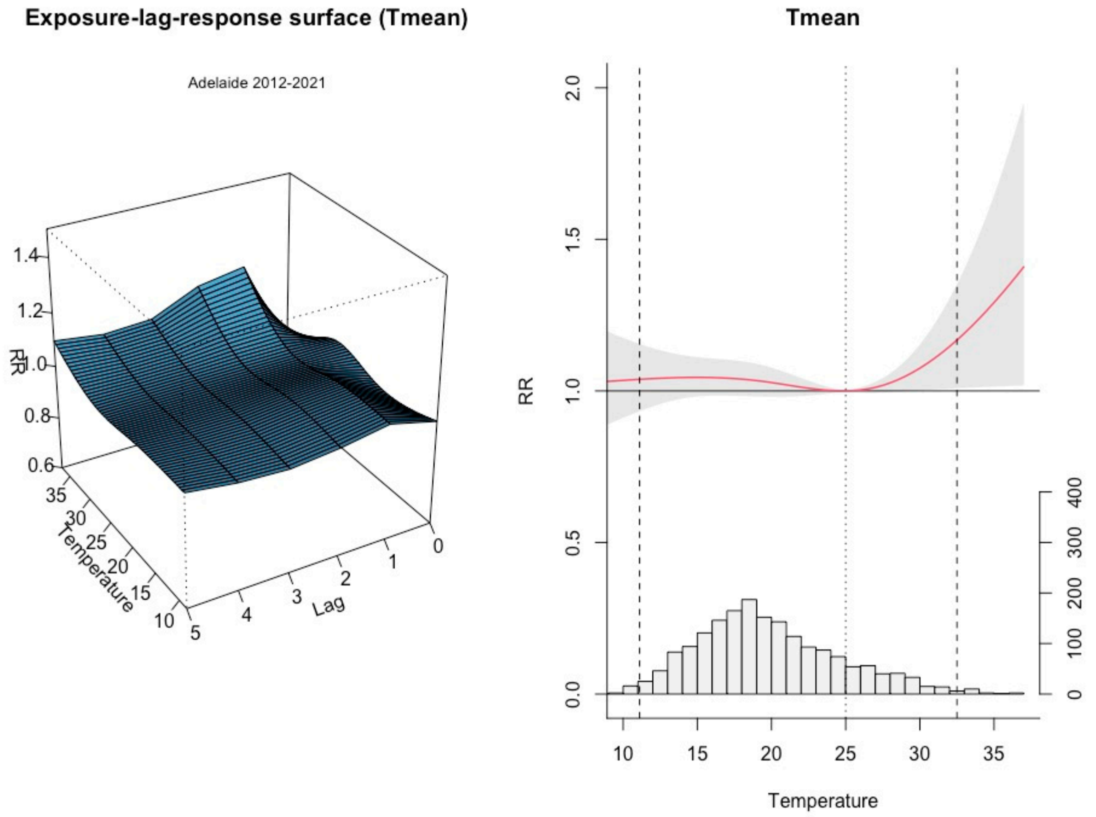

Figure S3. The three-dimensional plot of relationships between daily road crashes and daily mean temperature in Adelaide, 2012–2020 (left), and the overall cumulative exposure-response associations with mean temperature distributions (right). RR is the relative risk for road crashes.

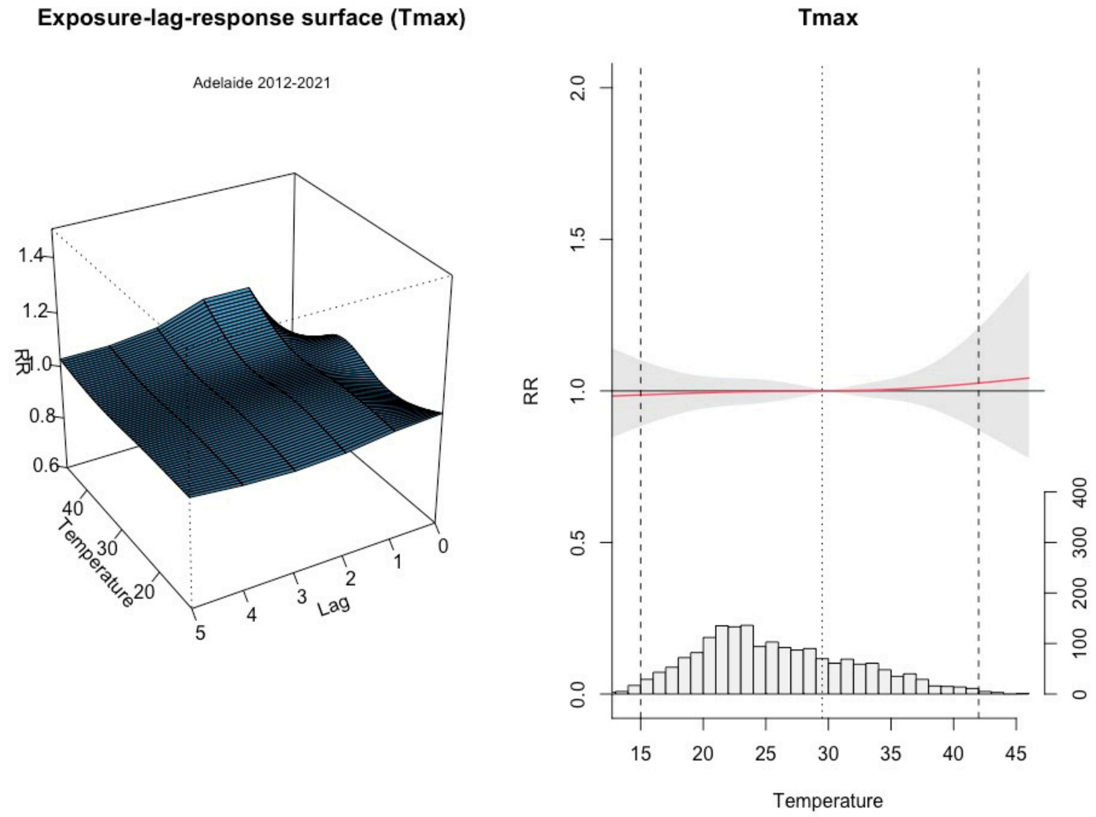

Figure S4. The three-dimensional plot of relationships between daily road crashes and daily maximum temperature in Adelaide, 2012–2020 (left), and the overall cumulative exposure-response associations with maximum temperature distributions (right). RR is the relative risk for road crashes.

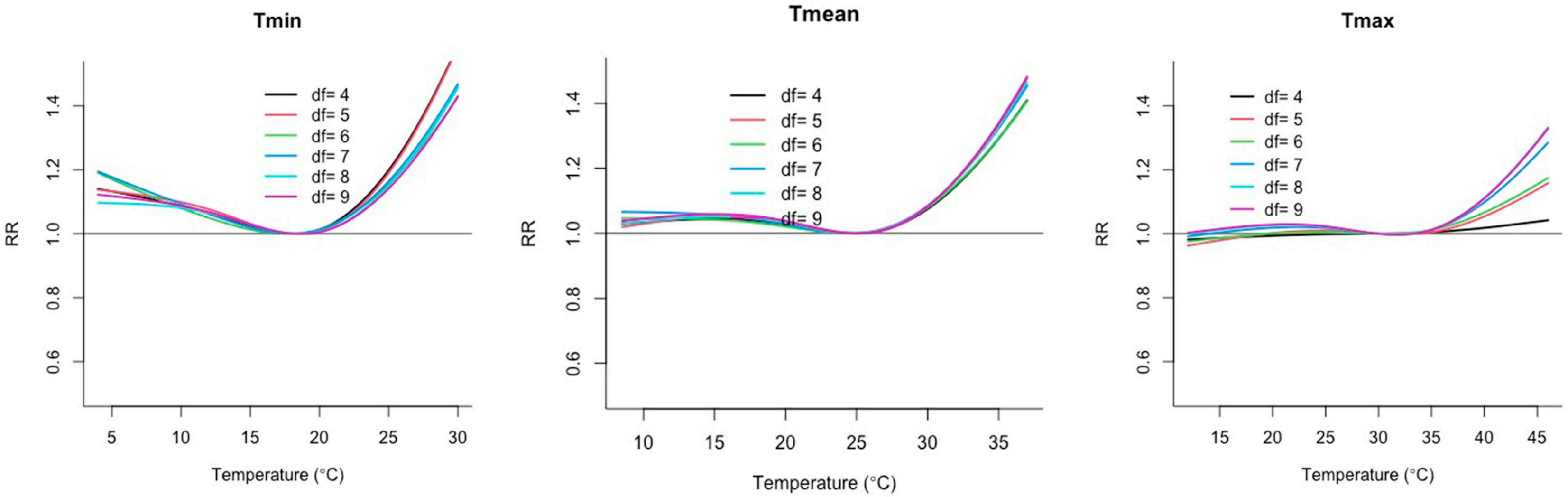

Figure S5. Overall cumulative exposure-response curve between temperatures (Tmin, Tmean and Tmax) and road crashes when changing df from 4 to 9 in Adelaide, South Australia 2012–2021. RR is the relative risk for road crashes.

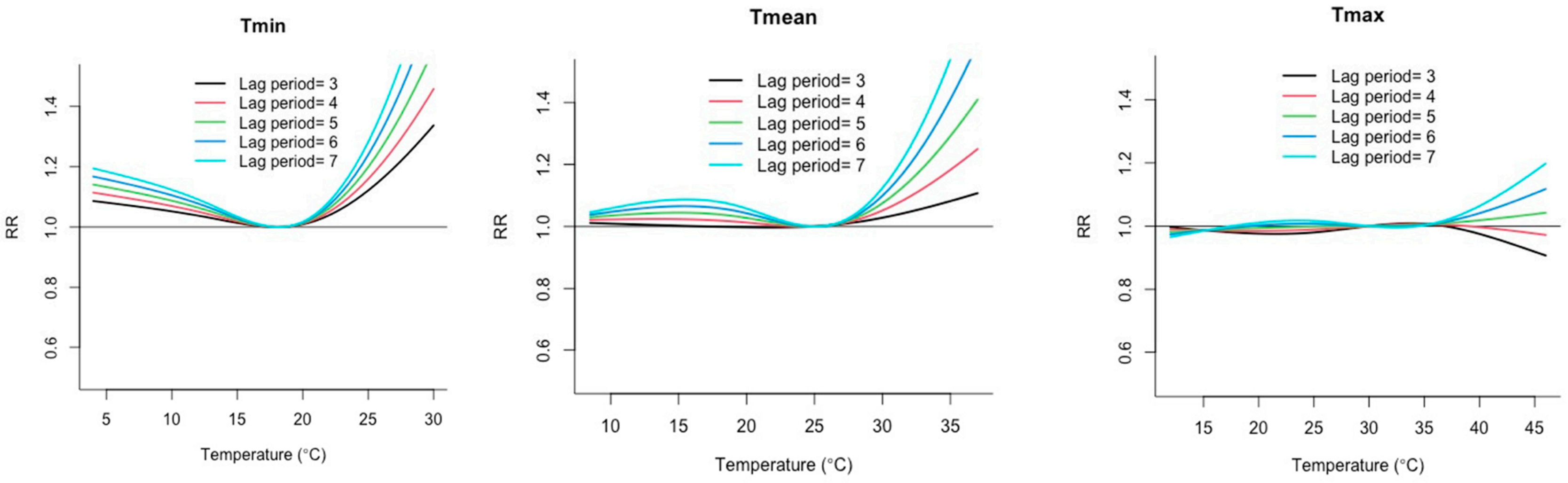

Figure S6. Overall cumulative exposure-response curve between temperatures (Tmin, Tmean and Tmax) and road crashes when changing the maximum lag days from 3 to 7 days in Adelaide, South Australia 2012–2021. RR is the relative risk for road crashes.

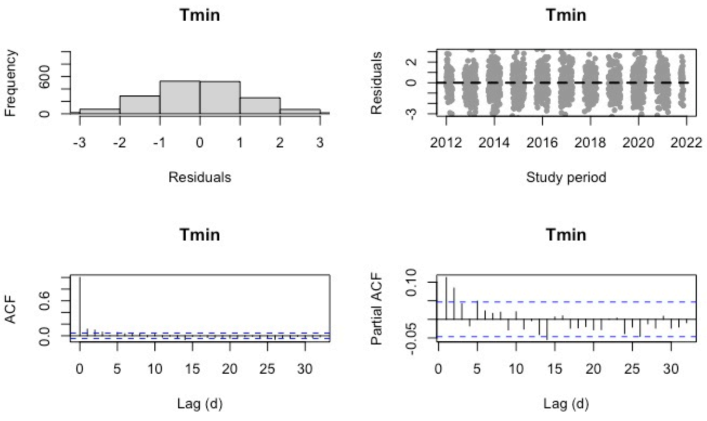

(**a**)

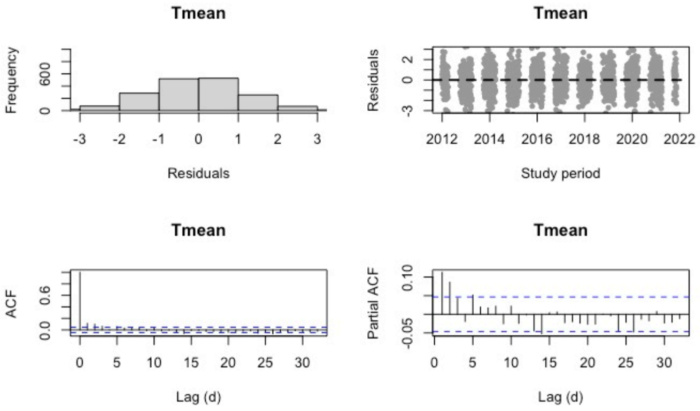

(**b**)

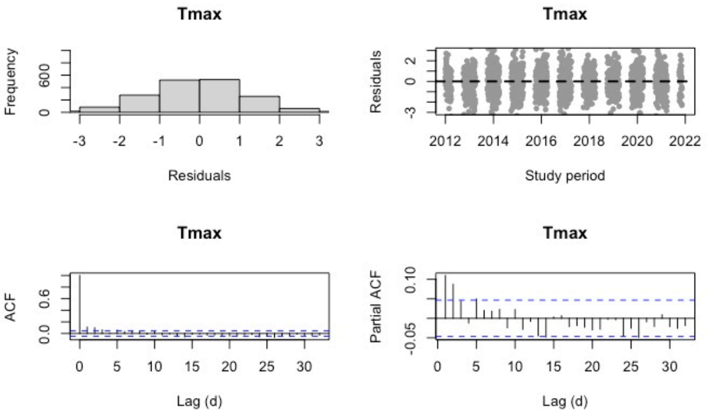

(**c**)

Figure S7. Histogram, Scatter plot, ACF (autocorrelation function) and PACF (partial autocorrelation function) plots of residuals using (**a**) Tmin, (**b**) Tmean and (**c**) Tmax derived from DLNM model for road crashes in Adelaide, South Australia 2012–2021.

Table S1. Results of the quasi-Poisson DLNM analysis of Tmin and road crashes in Adelaide, South Australia 2012–2021.

Coefficients: (2 not defined because of singularities)					
	Estimate	Std. Error	t value	Pr(>|t|)	
(Intercept)	-10.773664	0.163862	-65.748	< 2e-16	***
cb_tminv1.l1	-0.110169	0.050077	-2.2	0.027941	*
cb_tminv1.l2	0.011158	0.043593	0.256	0.798013	
cb_tminv1.l3	-0.042892	0.037157	-1.154	0.248528	
cb_tminv1.l4	-0.004551	0.033092	-0.138	0.890636	
cb_tminv2.l1	0.041582	0.130318	0.319	0.749703	
cb_tminv2.l2	0.105563	0.123943	0.852	0.394497	
cb_tminv2.l3	-0.004539	0.106831	-0.042	0.966116	
cb_tminv2.l4	-0.054148	0.090072	-0.601	0.547816	
cb_tminv3.l1	0.159778	0.10485	1.524	0.127729	
cb_tminv3.l2	0.041265	0.09941	0.415	0.678122	
cb_tminv3.l3	0.089506	0.088197	1.015	0.310325	
cb_tminv3.l4	-0.017858	0.072024	-0.248	0.804203	
a$beg_end	0.002554	0.025535	0.1	0.92035	
dow11	0.410124	0.021474	19.098	< 2e-16	***
dow12	0.482825	0.021191	22.784	< 2e-16	***
dow13	0.523142	0.021104	24.789	< 2e-16	***
dow14	0.536279	0.021009	25.527	< 2e-16	***
dow15	0.530983	0.020995	25.291	< 2e-16	***
dow16	0.20098	0.022391	8.976	< 2e-16	***
ns(a$rh1, df = 3)1	0.064501	0.029909	2.157	0.031177	*
ns(a$rh1, df = 3)2	0.320535	0.097961	3.272	0.001089	**
ns(a$rh1, df = 3)3	0.25196	0.062203	4.051	5.33826E-05	***
ns(a$radiation1, df = 3)1	0.112072	0.025063	4.472	8.27444E-06	***
ns(a$radiation1, df = 3)2	-0.067734	0.07986	-0.848	0.396468	
ns(a$radiation1, df = 3)3	0.092127	0.030399	3.031	0.002477	**
ns(a$daily_rain, df = 3)1	-0.060029	0.256031	-0.234	0.814656	
ns(a$daily_rain, df = 3)2	NA	NA	NA	NA	
ns(a$daily_rain, df = 3)3	NA	NA	NA	NA	
a$school_holidayperiods	-0.061599	0.023213	-2.654	0.008037	**
a$holiday_period	-0.321504	0.031853	-10.093	< 2e-16	***
ns(doy, df = dfseas)1:factor(year)2012	0.036978	0.215103	0.172	0.863529	
ns(doy, df = dfseas)2:factor(year)2012	0.167575	0.061323	2.733	0.006348	**
ns(doy, df = dfseas)3:factor(year)2012	1.094676	0.12884	8.496	< 2e-16	***
ns(doy, df = dfseas)4:factor(year)2012	-0.185782	0.082914	-2.241	0.025176	*
ns(doy, df = dfseas)1:factor(year)2013	0.034105	0.236017	0.145	0.885122	
ns(doy, df = dfseas)2:factor(year)2013	-0.014092	0.072416	-0.195	0.845735	
ns(doy, df = dfseas)3:factor(year)2013	0.335196	0.124726	2.687	0.00727	**
ns(doy, df = dfseas)4:factor(year)2013	-0.105047	0.097123	-1.082	0.27959	
ns(doy, df = dfseas)1:factor(year)2014	-0.099949	0.249733	-0.4	0.689043	
ns(doy, df = dfseas)2:factor(year)2014	0.005806	0.070823	0.082	0.934674	
ns(doy, df = dfseas)3:factor(year)2014	0.434366	0.118486	3.666	0.000254	***
ns(doy, df = dfseas)4:factor(year)2014	-0.096955	0.098114	-0.988	0.323201	
ns(doy, df = dfseas)1:factor(year)2015	0.368941	0.238523	1.547	0.122103	
ns(doy, df = dfseas)2:factor(year)2015	-0.001065	0.07144	-0.015	0.988106	
ns(doy, df = dfseas)3:factor(year)2015	0.410102	0.114604	3.578	0.000355	***
ns(doy, df = dfseas)4:factor(year)2015	-0.023276	0.100038	-0.233	0.816042	
ns(doy, df = dfseas)1:factor(year)2016	-0.16316	0.247284	-0.66	0.509466	
ns(doy, df = dfseas)2:factor(year)2016	0.015862	0.069598	0.228	0.819741	
ns(doy, df = dfseas)3:factor(year)2016	1.124563	0.113761	9.885	< 2e-16	***
ns(doy, df = dfseas)4:factor(year)2016	0.69619	0.083313	8.356	< 2e-16	***
ns(doy, df = dfseas)1:factor(year)2017	0.04914	0.271672	0.181	0.856482	
ns(doy, df = dfseas)2:factor(year)2017	-0.0388	0.078324	-0.495	0.620401	
ns(doy, df = dfseas)3:factor(year)2017	0.320602	0.123187	2.603	0.009333	**
ns(doy, df = dfseas)4:factor(year)2017	0.027032	0.103656	0.261	0.794287	
ns(doy, df = dfseas)1:factor(year)2018	0.307302	0.256531	1.198	0.231117	
ns(doy, df = dfseas)2:factor(year)2018	-0.015607	0.07898	-0.198	0.84338	
ns(doy, df = dfseas)3:factor(year)2018	0.459992	0.12791	3.596	0.000332	***
ns(doy, df = dfseas)4:factor(year)2018	0.005928	0.103659	0.057	0.954401	
ns(doy, df = dfseas)1:factor(year)2019	0.017305	0.258302	0.067	0.946594	
ns(doy, df = dfseas)2:factor(year)2019	0.098078	0.080015	1.226	0.220466	
ns(doy, df = dfseas)3:factor(year)2019	0.4286	0.135953	3.153	0.001647	**

ns(doy, df = dfseas)4:factor(year)2019	-0.231397	0.10769	-2.149	0.031796	*
ns(doy, df = dfseas)1:factor(year)2020	-0.102311	0.280435	-0.365	0.715284	
ns(doy, df = dfseas)2:factor(year)2020	0.116222	0.080094	1.451	0.146943	
ns(doy, df = dfseas)3:factor(year)2020	0.30109	0.149124	2.019	0.043637	*
ns(doy, df = dfseas)4:factor(year)2020	-0.064186	0.104793	-0.612	0.540291	
ns(doy, df = dfseas)1:factor(year)2021	-0.629157	0.264404	-2.38	0.017444	*
ns(doy, df = dfseas)2:factor(year)2021	0.232051	0.079343	2.925	0.003494	**
ns(doy, df = dfseas)3:factor(year)2021	0.916273	0.156499	5.855	5.72E-09	***
ns(doy, df = dfseas)4:factor(year)2021	-0.349252	0.102652	-3.402	0.000684	***

Signif. codes: 0 '***' 0.001 '**' 0.01 '*' 0.05 '.' 0.1 ' ' 1

Table S2. Results of the quasi-Poisson DLNM analysis of Tmean and road crashes in Adelaide, South Australia 2012–2021.

Coefficients: (2 not defined because of singularities)

	Estimate	Std. Error	t value	Pr(>|t|)	
(Intercept)	-10.8338264	0.1646347	-65.805	< 2e-16	***
cb2v1.l1	-0.1492264	0.054673	-2.729	0.00641	**
cb2v1.l2	-0.0006287	0.0446055	-0.014	0.988757	
cb2v1.l3	0.050467	0.0388975	1.297	0.194658	
cb2v1.l4	-0.0816614	0.033996	-2.402	0.016408	*
cb2v2.l1	0.1568463	0.1677901	0.935	0.350036	
cb2v2.l2	0.1520169	0.1524921	0.997	0.318963	
cb2v2.l3	-0.0757816	0.1267704	-0.598	0.550062	
cb2v2.l4	0.1123561	0.1057747	1.062	0.288286	
cb2v3.l1	0.2435826	0.1134439	2.147	0.031921	*
cb2v3.l2	0.0924584	0.1030112	0.898	0.369549	
cb2v3.l3	-0.0529182	0.0973038	-0.544	0.586619	
cb2v3.l4	0.1339603	0.072972	1.836	0.066565	.
a$beg_end	0.0037585	0.0254574	0.148	0.882645	
dow11	0.4089242	0.0213968	19.111	< 2e-16	***
dow12	0.4815426	0.0211015	22.82	< 2e-16	***
dow13	0.522424	0.0210025	24.874	< 2e-16	***
dow14	0.536542	0.020928	25.637	< 2e-16	***
dow15	0.5299874	0.0209166	25.338	< 2e-16	***
dow16	0.2018596	0.0222907	9.056	< 2e-16	***
ns(a$rh1, df = 3)1	0.0758634	0.034423	2.204	0.027668	*
ns(a$rh1, df = 3)2	0.2704563	0.1064478	2.541	0.01115	*
ns(a$rh1, df = 3)3	0.248246	0.0630883	3.935	8.65789E-05	***
ns(a$radiation1, df = 3)1	0.1136276	0.0245569	4.627	3.98825E-06	***
ns(a$radiation1, df = 3)2	-0.0665468	0.0788695	-0.844	0.398923	
ns(a$radiation1, df = 3)3	0.0956977	0.0294279	3.252	0.001169	**
ns(a$daily_rain, df = 3)1	-0.0730755	0.2536627	-0.288	0.77332	
ns(a$daily_rain, df = 3)2	NA	NA	NA	NA	
ns(a$daily_rain, df = 3)3	NA	NA	NA	NA	
a$school_holidayperiods	-0.0690042	0.0231495	-2.981	0.002916	**
a$holiday_period	-0.3264425	0.0317007	-10.298	< 2e-16	***
ns(doy, df = dfseas)1:factor(year)2012	0.1206361	0.2077701	0.581	0.561571	
ns(doy, df = dfseas)2:factor(year)2012	0.1799218	0.0610774	2.946	0.003265	**
ns(doy, df = dfseas)3:factor(year)2012	1.0967948	0.1283544	8.545	< 2e-16	***
ns(doy, df = dfseas)4:factor(year)2012	-0.1643333	0.0822677	-1.998	0.045925	*
ns(doy, df = dfseas)1:factor(year)2013	0.0811635	0.2345397	0.346	0.729344	
ns(doy, df = dfseas)2:factor(year)2013	0.0076391	0.0715102	0.107	0.91494	
ns(doy, df = dfseas)3:factor(year)2013	0.3319739	0.1248034	2.66	0.007888	**
ns(doy, df = dfseas)4:factor(year)2013	-0.1038075	0.0970034	-1.07	0.284707	
ns(doy, df = dfseas)1:factor(year)2014	0.0351078	0.2462381	0.143	0.886641	
ns(doy, df = dfseas)2:factor(year)2014	0.0031065	0.0708851	0.044	0.96505	
ns(doy, df = dfseas)3:factor(year)2014	0.4141909	0.1192372	3.474	0.000526	***
ns(doy, df = dfseas)4:factor(year)2014	-0.0592532	0.0978491	-0.606	0.54489	
ns(doy, df = dfseas)1:factor(year)2015	0.4217445	0.2361363	1.786	0.074274	.
ns(doy, df = dfseas)2:factor(year)2015	0.001241	0.0712067	0.017	0.986097	
ns(doy, df = dfseas)3:factor(year)2015	0.4075731	0.1142075	3.569	0.000369	***
ns(doy, df = dfseas)4:factor(year)2015	-0.0175838	0.09972	-0.176	0.860055	
ns(doy, df = dfseas)1:factor(year)2016	-0.115741	0.2501267	-0.463	0.643617	

ns(doy, df = dfseas)2:factor(year)2016	0.0488778	0.0679832	0.719	0.472258	
ns(doy, df = dfseas)3:factor(year)2016	1.1206333	0.1148377	9.758	< 2e-16	***
ns(doy, df = dfseas)4:factor(year)2016	0.7075681	0.0831407	8.51	< 2e-16	***
ns(doy, df = dfseas)1:factor(year)2017	0.1393898	0.2663762	0.523	0.600846	
ns(doy, df = dfseas)2:factor(year)2017	-0.0303579	0.078089	-0.389	0.697502	
ns(doy, df = dfseas)3:factor(year)2017	0.2965388	0.1233867	2.403	0.016353	*
ns(doy, df = dfseas)4:factor(year)2017	0.0478214	0.1030754	0.464	0.642746	
ns(doy, df = dfseas)1:factor(year)2018	0.3428515	0.2553034	1.343	0.179478	
ns(doy, df = dfseas)2:factor(year)2018	0.0028516	0.0778103	0.037	0.97077	
ns(doy, df = dfseas)3:factor(year)2018	0.4443337	0.1278134	3.476	0.000521	***
ns(doy, df = dfseas)4:factor(year)2018	0.0022336	0.1034457	0.022	0.982776	
ns(doy, df = dfseas)1:factor(year)2019	0.0476245	0.2571845	0.185	0.853113	
ns(doy, df = dfseas)2:factor(year)2019	0.1249845	0.0795434	1.571	0.116304	
ns(doy, df = dfseas)3:factor(year)2019	0.4202688	0.1359414	3.092	0.002023	**
ns(doy, df = dfseas)4:factor(year)2019	-0.2191428	0.1080925	-2.027	0.042781	*
ns(doy, df = dfseas)1:factor(year)2020	0.0015871	0.2770494	0.006	0.99543	
ns(doy, df = dfseas)2:factor(year)2020	0.1132277	0.0798492	1.418	0.156368	
ns(doy, df = dfseas)3:factor(year)2020	0.3068217	0.1493365	2.055	0.040073	*
ns(doy, df = dfseas)4:factor(year)2020	-0.0216068	0.1037966	-0.208	0.835125	
ns(doy, df = dfseas)1:factor(year)2021	-0.5534132	0.2612195	-2.119	0.034271	*
ns(doy, df = dfseas)2:factor(year)2021	0.2436799	0.0793046	3.073	0.002155	**
ns(doy, df = dfseas)3:factor(year)2021	0.9141996	0.1564013	5.845	6.05E-09	***
ns(doy, df = dfseas)4:factor(year)2021	-0.3354627	0.1022241	-3.282	0.001053	**

Signif. codes: 0 '***' 0.001 '**' 0.01 '*' 0.05 '.' 0.1 ' ' 1

Table S3. Results of the quasi-Poisson DLNM analysis of Tmax and road crashes in Adelaide, South Australia 2012–2021.

Coefficients: (2 not defined because of singularities)

	Estimate	Std. Error	t value	Pr(>|t|)	
(Intercept)	-10.8283486	0.1644542	-65.844	< 2e-16	***
cb_tmaxv1.l1	-0.1040512	0.0464653	-2.239	0.025263	*
cb_tmaxv1.l2	-0.0251566	0.0401257	-0.627	0.530779	
cb_tmaxv1.l3	0.0868067	0.0357927	2.425	0.015401	*
cb_tmaxv1.l4	-0.0671753	0.0306669	-2.19	0.028624	*
cb_tmaxv2.l1	0.1817901	0.1517527	1.198	0.231108	
cb_tmaxv2.l2	0.1859217	0.1406051	1.322	0.186247	
cb_tmaxv2.l3	-0.1754066	0.1203784	-1.457	0.145266	
cb_tmaxv2.l4	0.141872	0.0981222	1.446	0.148398	
cb_tmaxv3.l1	0.2010742	0.1027045	1.958	0.050417	.
cb_tmaxv3.l2	0.1042146	0.0946848	1.101	0.271206	
cb_tmaxv3.l3	-0.153914	0.0867159	-1.775	0.076089	.
cb_tmaxv3.l4	0.1243325	0.0647669	1.92	0.055064	.
a$beg_end	0.0001046	0.025522	0.004	0.996729	
dow11	0.4085194	0.0214162	19.075	< 2e-16	***
dow12	0.4816055	0.0211228	22.8	< 2e-16	***
dow13	0.5235233	0.0210254	24.9	< 2e-16	***
dow14	0.5363241	0.0209519	25.598	< 2e-16	***
dow15	0.5294167	0.0209428	25.279	< 2e-16	***
dow16	0.2030028	0.0223131	9.098	< 2e-16	***
ns(a$rh1, df = 3)1	0.0797788	0.0341607	2.335	0.019638	*
ns(a$rh1, df = 3)2	0.2270415	0.1031034	2.202	0.027794	*
ns(a$rh1, df = 3)3	0.2324591	0.0631541	3.681	0.00024	***
ns(a$radiation1, df = 3)1	0.1120711	0.0251728	4.452	9.05679E-06	***
ns(a$radiation1, df = 3)2	-0.0868143	0.079885	-1.087	0.277305	
ns(a$radiation1, df = 3)3	0.0881885	0.0307171	2.871	0.004142	**
ns(a$daily_rain, df = 3)1	-0.0722357	0.2551355	-0.283	0.777114	
ns(a$daily_rain, df = 3)2	NA	NA	NA	NA	
ns(a$daily_rain, df = 3)3	NA	NA	NA	NA	
a$school_holidayperiods	-0.0665431	0.0229914	-2.894	0.003849	**
a$holiday_period	-0.3271197	0.031671	-10.329	< 2e-16	***
ns(doy, df = dfseas)1:factor(year)2012	0.1459455	0.2014558	0.724	0.468887	
ns(doy, df = dfseas)2:factor(year)2012	0.1813415	0.0612923	2.959	0.003133	**
ns(doy, df = dfseas)3:factor(year)2012	1.0909679	0.1287244	8.475	< 2e-16	***
ns(doy, df = dfseas)4:factor(year)2012	-0.168442	0.0822141	-2.049	0.040633	*

ns(doy, df = dfseas)1:factor(year)2013	0.0941764	0.2339223	0.403	0.687295	
ns(doy, df = dfseas)2:factor(year)2013	0.016941	0.0711141	0.238	0.811737	
ns(doy, df = dfseas)3:factor(year)2013	0.3340005	0.1252938	2.666	0.007755	**
ns(doy, df = dfseas)4:factor(year)2013	-0.0986403	0.0972352	-1.014	0.310512	
ns(doy, df = dfseas)1:factor(year)2014	0.0262497	0.2421317	0.108	0.913683	
ns(doy, df = dfseas)2:factor(year)2014	0.0026377	0.0708372	0.037	0.970301	
ns(doy, df = dfseas)3:factor(year)2014	0.4213092	0.118793	3.547	0.000401	***
ns(doy, df = dfseas)4:factor(year)2014	-0.0700757	0.0977198	-0.717	0.473406	
ns(doy, df = dfseas)1:factor(year)2015	0.4141935	0.2354318	1.759	0.078707	.
ns(doy, df = dfseas)2:factor(year)2015	0.0001755	0.0712387	0.002	0.998035	
ns(doy, df = dfseas)3:factor(year)2015	0.4243889	0.1142363	3.715	0.00021	***
ns(doy, df = dfseas)4:factor(year)2015	-0.0031954	0.0998824	-0.032	0.974482	
ns(doy, df = dfseas)1:factor(year)2016	-0.0988273	0.2489434	-0.397	0.691427	
ns(doy, df = dfseas)2:factor(year)2016	0.0722723	0.0674451	1.072	0.284065	
ns(doy, df = dfseas)3:factor(year)2016	1.0972754	0.1163443	9.431	< 2e-16	***
ns(doy, df = dfseas)4:factor(year)2016	0.7091696	0.0833064	8.513	< 2e-16	***
ns(doy, df = dfseas)1:factor(year)2017	0.145355	0.2637938	0.551	0.581694	
ns(doy, df = dfseas)2:factor(year)2017	-0.0168515	0.0781743	-0.216	0.829354	
ns(doy, df = dfseas)3:factor(year)2017	0.2727508	0.1242557	2.195	0.028293	*
ns(doy, df = dfseas)4:factor(year)2017	0.0550254	0.103018	0.534	0.593319	
ns(doy, df = dfseas)1:factor(year)2018	0.3265861	0.2550424	1.281	0.200538	
ns(doy, df = dfseas)2:factor(year)2018	0.0165399	0.0775619	0.213	0.831159	
ns(doy, df = dfseas)3:factor(year)2018	0.4380186	0.1285022	3.409	0.000668	***
ns(doy, df = dfseas)4:factor(year)2018	-0.0037977	0.1036002	-0.037	0.970763	
ns(doy, df = dfseas)1:factor(year)2019	0.067438	0.257214	0.262	0.79321	
ns(doy, df = dfseas)2:factor(year)2019	0.1287249	0.0793951	1.621	0.105134	
ns(doy, df = dfseas)3:factor(year)2019	0.4300809	0.1357623	3.168	0.001563	**
ns(doy, df = dfseas)4:factor(year)2019	-0.1842666	0.1084199	-1.7	0.089396	.
ns(doy, df = dfseas)1:factor(year)2020	0.0243706	0.2735155	0.089	0.929012	
ns(doy, df = dfseas)2:factor(year)2020	0.1120987	0.080229	1.397	0.162525	
ns(doy, df = dfseas)3:factor(year)2020	0.3003067	0.1497182	2.006	0.045034	*
ns(doy, df = dfseas)4:factor(year)2020	-0.020427	0.1035628	-0.197	0.843661	
ns(doy, df = dfseas)1:factor(year)2021	-0.5349535	0.258316	-2.071	0.038516	*
ns(doy, df = dfseas)2:factor(year)2021	0.2531716	0.0795841	3.181	0.001493	**
ns(doy, df = dfseas)3:factor(year)2021	0.9089969	0.156753	5.799	7.94E-09	***
ns(doy, df = dfseas)4:factor(year)2021	-0.3367307	0.102293	-3.292	0.001016	**

Signif. codes: 0 '***' 0.001 '**' 0.01 '*' 0.05 '.' 0.1 ' ' 1
